# Dysfunctional Natural Killer Cells in the Aftermath of Cancer Surgery

**DOI:** 10.3390/ijms18081787

**Published:** 2017-08-17

**Authors:** Leonard Angka, Sarwat T. Khan, Marisa K. Kilgour, Rebecca Xu, Michael A. Kennedy, Rebecca C. Auer

**Affiliations:** 1 Centre for Innovative Cancer Research, Ottawa Hospital Research Institute, Ottawa, ON K1H 8L6, Canada; langk069@uottawa.ca (L.A.); skhan177@uottawa.ca (S.T.K.); rxu069@uottawa.ca (R.X.); michaelkennedy.ohri@gmail.com (M.A.K.); 2 Department of Biochemistry, Microbiology and Immunology, University of Ottawa, Ottawa, ON K1H 8M5, Canada; 3 Deeley Research Centre, BC Cancer Agency, Victoria, BC V8R 6V5, Canada; mkilg052@uottawa.ca; 4 Department of Surgery, University of Ottawa, Ottawa, ON K1H 8L6, Canada

**Keywords:** Natural Killer (NK) cells, surgery, immunosuppression, peri-operative therapies, immunity, immunotherapy

## Abstract

The physiological changes that occur immediately following cancer surgeries initiate a chain of events that ultimately result in a short pro-, followed by a prolonged anti-, inflammatory period. Natural Killer (NK) cells are severely affected during this period in the recovering cancer patient. NK cells play a crucial role in anti-tumour immunity because of their innate ability to differentiate between malignant versus normal cells. Therefore, an opportunity arises in the aftermath of cancer surgery for residual cancer cells, including distant metastases, to gain a foothold in the absence of NK cell surveillance. Here, we describe the post-operative environment and how the release of sympathetic stress-related factors (e.g., cortisol, prostaglandins, catecholamines), anti-inflammatory cytokines (e.g., IL-6, TGF-β), and myeloid derived suppressor cells, mediate NK cell dysfunction. A snapshot of current and recently completed clinical trials specifically addressing NK cell dysfunction post-surgery is also discussed. In collecting and summarizing results from these different aspects of the surgical stress response, a comprehensive view of the NK cell suppressive effects of surgery is presented. Peri-operative therapies to mitigate NK cell suppression in the post-operative period could improve curative outcomes following cancer surgery.

## 1. Introduction

Over a century of controversial reporting on the adverse sequelae of cancer surgery is found throughout scientific literature [1,2,3,4,5]. While the immediate benefits of solid tumour resections are indisputable, the unintended adverse consequences of surgery pose a serious concern for patient recovery, residual tumour control, and relapse free survival. An early retrospective study of 1173 breast cancer patients undergoing mastectomy alone was compared to a historical [6] dataset of 250 untreated breast cancer patients for death specific hazard rates [3]. Demicheli and colleagues found a sobering difference between these two datasets as the patients receiving mastectomies had two hazard rate peaks (at the third and eighth year), while those untreated patients only had one peak (at the fourth year). These results indicate that surgery for breast cancer treatment accelerated mortality for a subset of patients as compared to those not receiving any treatment at all. Furthermore, in numerous preclinical animal studies, surgical stress has been shown to enhance metastases and reduce survival [7,8,9,10,11,12,13].

Multiple physiological processes are altered by surgical stress and compelling reports show that invasive procedures accelerate residual tumour growth and metastases through profound suppression of the immune system [14,15,16,17,18]. Coffey et al. describes the “immunological window of opportunity” following surgery which may make the patient more susceptible to tumour growth and metastases as cellular immunity is impaired [4].

The pivotal role Natural Killer (NK) cells play in establishing anti-tumour immunity is severely suppressed in the wake of surgery and persists for weeks after [17]. In this review, we describe the evidence supporting how the inflammatory response to surgical stress impacts NK cell anti-tumour surveillance including findings from current clinical trials attempting to combat post-operative NK cell suppression. Cancer immunotherapy has provided powerful methods for appropriating the immune system to combat disease. However, despite the recognition that post-operative immune suppression contributes to disease recurrence, the peri-operative window remains unutilized with no FDA approved therapeutic drug available for cancer patients to alleviate the deleterious effects of immune suppression after surgery [19].

## 2. Natural Killer Cell Anti-Tumour Immunity

NK cells were first identified by Kiessling et al. in 1975 [20] from the observation that red-blood-cell-lysed splenocytes are able to naturally kill Moloney leukemia cells without prior in vivo sensitization. Stemming from these initial findings, we have now come to define NK cells as licensed cytolytic cells of the innate immune system which stand guard for atypical cell surface antigen-ligands on stressed, infected or cancerous cells [21]. Importantly, our current understanding of NK cell cytotoxicity posits that a balance between activating and inhibitory stimuli regulate NK cell activation and thus prevent non-specific killing of healthy tissues. Multiple inhibitory receptors, such as, the killer Ig-like receptors (KIRs) and the CD94-NKG2A heterodimer are expressed on the surface of NK cells, which recognize HLA-class I and HLA-E molecules respectively, have been described [22,23]. These inhibitory receptors enable target cell recognition and also play a key role in the licensing/education of NK cells during NK cell development [24,25]. NK cells without self-MHC inhibitory receptors are hyporesponsive whereas NK cells with inhibitory KIRs for self-MHC have increased cytotoxicity, adhesion, and effector functions against targets [26,27]. Therefore, NK cells are especially effective at controlling tumours since tumours downregulate their MHC class I expression during immunoediting to escape CD8^+^ T cells. However, the absence of MHC-I alone is not sufficient to activate NK cells [27].

Engagement of activating receptors present on NK cells, such as NKG2D and the natural cytoxicity receptors (NCRs) NKp46, NKp44, and NKp30, is also required to initiate downstream cytotoxic mechanisms [28]. Although the ligands for these receptors are poorly defined and a topic of considerable interest, recent work has determined that NKG2D recognizes cell surface glycoproteins (including MHC-class I polypeptide-related sequence A (MICA), MICB, UL16-binding proteins 1–6 (ULPBs)) and NCRs bind heparin sulfate [29,30]. Importantly, these activating ligands are upregulated on various tumour cells types making them susceptible to NK cell targeting. Thus, NK cell surveillance is especially effective at detecting and destroying nascent tumours as they have reduced MHC-I expression and express ligands for NK activating receptors. Upon target identification, NK cells release cytotoxic granules containing perforin and granzymes to initiate an apoptotic signaling cascade in target cells [31]. In addition to their cytotoxic effector functions, NK cells can also be stimulated to produce IFNγ and TNFα in response to various cytokines such as interleukin (IL)-2, IL-12, IL-15, and IL-18.

NK cells are critical for anti-tumour immunity. In a prospective study conducted by Imai and colleagues, a cohort of 154 people (>40 years old) were followed up and assessed for associations between peripheral blood lymphocyte (PBL) cytotoxicity and cancer incidence after 11 years [32]. Using a NK cell specific target leukemia cell line, K562, a standard chromium-51 (Cr-51) release assay was performed with PBL. They reported that medium to high cytotoxicity of PBLs against K562 was associated with significantly reduced risk of cancer. In addition to highlighting the importance of NK cell function in determining cancer incidence, the results of this study also suggest that within a given population there are individuals who have a predisposition to better or worse NK cell activity. In a subsequent study, the authors reported that high cytotoxicity of NK cells can be traced back to a set of single nucleotide polymorphisms (SNPs) found in the natural killer complex (NKC) on chromosome 12p, with the majority of SNPs located in the *NKG2D* gene region [33]. The high NK activity haplotype (HNK1/HNK1) strongly correlated with NK cell cytotoxicity and the reduced risk of colorectal cancer (CRC) [33,34]. Furthermore, multiple reports have shown that either infiltration of NK cells [35,36] or increased expression of NKG2D ligands (MIC or ULPBs) [37] can be used as a prognostic marker in various carcinomas. These findings together clearly underscore the importance of NK cells in establishing anti-tumour immunity.

## 3. Natural Killer Cell Dysfunction after Surgery

The physiological toll and consequences of surgery has direct effects on NK cell function. A study by Iannone et al. [38] showed that NK cell cytotoxicity is significantly decreased (*p* = < 0.005) following surgery on post-operation day (POD) 7 in 24 pancreatic cancer patients undergoing duodenopancreatectomies which was not dependent on NK cell number. By POD30, NK cell cytotoxicity was restored [38]. We have reported a similar phenomenon in CRC surgery patient peripheral blood mononuclear cells (PBMCs) collected before surgery, on POD1, POD3, and POD28. NK cell cytotoxicity is severely suppressed on POD1 (>70% compared to baseline) and gradually returns to pre-operative levels by POD28 [7]. Velasquez et al. [39] reported that even in rarer cancer surgeries such as primary bone cancer, patient’s NK cells are significantly reduced post-operatively in their ability to kill target K562 leukemia cells for up to five days after surgery. These studies show that NK cell cytotoxicity is severely affected following surgery for up to seven days but recover by POD28.

NK cell cytotoxicity is not the only mechanism that is disrupted by surgical stress. It has been reported that on POD1 NK cells suffer a marked reduction in their cytokine secretion in response to stimulation. CD3^−^CD56^bright^CD16^neg^ NK cell subsets are regarded as the main cytokine producing NK cells (CD56^bright^). Reinhardt et al. [40] showed that peripheral blood CD56^bright^ NK cells have exceptionally reduced IFNγ production when stimulated with *staphylococcus aureus* or recombinant IL-12 on POD1. They observed a significant decrease in IL-12R on CD56^bright^ NK cells, however this did not affect the pSTAT4 levels in post-operative surgery patients which is involved in the transcriptional regulation of IFNγ [41]. Furthermore, neutralizing antibodies against IL-10 did not restore post-operative IFNγ production which, when taken together with the observation that pSTAT4 levels were not affected indicates that a cytokine-independent affliction is altering NK cell cytokine secretion [40]. Research from our lab extends these findings as we have seen an inability of NK cell stimulation from whole blood which persists for up to four weeks after surgery in CRC patients [42]. It is curious that IFNγ production is suppressed for multiple weeks post-surgery while NK cell cytotoxicity appears to recover within one week. Whether this is the result of how these parameters are experimentally collected or a deeper reflection of specific pathways being suppressed in response to surgery is currently under investigation by our lab.

From these studies it is clear that the mechanisms responsible for NK cell dysfunction can persist for weeks after surgery which results in a reduced anti-tumour surveillance period in which residual cancers can progress and metastasize. What physiological events are taking place during the post-operative period which results in such a dramatic reduction in NK cell effector mechanisms? The remainder of this review will describe the changes that ensue immediately following surgery and how they may drastically effect NK cell functions.

## 4. Surgical Stress, Inflammation and Natural Killer Cell Dysfunction

The inflammatory response to surgery is made up of two phases—an acute pro-inflammatory phase followed by a prolonged anti-inflammatory phase [43]. Briefly, inflammation at the wound or surgical site is first initiated by the release of stress signals known as “damage-associated molecular patterns” (DAMPs) which can be sensed by pattern recognition receptors (PRRs) on monocytes. Monocyte PRRs (e.g., toll-like receptors, C-type lectin receptors) bind to DAMPs (e.g., HMGB1, S100 proteins, actin, HSP60 and HSP70) which leads to the large production of pro-inflammatory cytokines (e.g., IL-1β, IL-6, and TNF) through NFκB activity. IL-6 has dual pro- and anti-inflammatory roles [44], but following the acute pro-inflammatory phase the prevailing anti-inflammatory properties of IL-6 set the stage for the next phase of surgical inflammation. The anti-inflammatory phase is characterized by: the negative feedback of IL-1 and TNFα by IL-6; the accumulation of C-reactive protein (CRP); the downregulation of HLA-DR expression on monocytes and; the induction of IL-10 through prostaglandin E2 (PGE2) [18,43,45,46]. For these reasons, IL-6 has been used in many studies as an indicator of surgical stress.

In a study comparing the peri-operative serum cytokine levels of prostate cancer surgery patients undergoing either a laparoscopic radical prostectomy (LRP; *n* = 66) or an open radical prostectomy (ORP; *n* = 99), serum IL-6 levels increased immediately after operation in all surgery groups. However, the LRP (less invasive surgical procedure) cohort serum IL-6 levels were significantly lower than the ORP cohort (68 pg/mL vs. 135.4 pg/mL; *p* = 0.006), on POD 1, indicating a lesser surgical insult [47]. A similar trend was true for IL-1β, IL-10 and CRP in these patients [47]. Although many factors are involved in determining the strength of the inflammatory response to surgery, the nature of the surgical procedure, site, length, underlying disease, and patient demographics (age, cancer stage, ethnicity, lifestyle) are major determinants. Strong evidence supports that the degree of induced stress and the invasive nature of the procedure are correlated with the degree of immune suppression post-surgery. A study comparing robotic, laparoscopic and open surgery on colorectal resection patients (with primary tumours) revealed significant decreases in markers of immune competence (HLA-DR expression on monocytes) post-surgery [48]. As expected, levels of CRP were higher in the open surgery group further corroborating that increased surgical stress increases immune suppression.

### 4.1. Consequences of Surgical Inflammation on NK Cells

The post-operative cytokine milieu is dominated by IL-6 in both the peripheral blood, but even more so at the site of surgical trauma. Decker et al. [49] showed that drainage fluid from abdominal surgeries had a 30-fold increase in IL-6 on POD1 while peripheral blood IL-6 was increased by 7.4-fold compared to pre-operative levels. The immunomodulatory effect of IL-6 on NK cell function has been scarcely reported on. The results from a Phase I clinical study in 20 patients with advanced cancer (colon and pancreatic) receiving recombinant IL-6 (rIL-6) showed the suppressive effects of rIL-6 on NK cell cytotoxicity [50]. Patients given rIL-6 suffered a 50% drop in NK cell cytotoxicity on day 7 after treatment which gradually returned by day 20. A second administration of rIL-6 at day 22 also decreased NK cell cytotoxicity on day 30 in the same patients showing a clear effect of rIL-6 administration on NK cell function [50]. Recent studies have demonstrated that IL-6 inhibits the cytotoxic activity of NK cells by decreasing perforin and granzyme production by modulating SHP-2 expression [51,52,53]. Cifaldi and colleagues [52] reported that adding rIL-6 to human NK cell cultures significantly reduces expression of perforin and granzyme B. However, blocking IL-6 signaling with tocilizumab or soluble IL-6R restored perforin and granzyme B expression and also significantly improved NK cell cytotoxicity [52]. Together these studies show a possible direct role of IL-6 on NK cell function.

IL-6 can also lead to the production of PGE2 which has been reported to have direct effects on NK cells [54]. Two day cultures of isolated human NK cells with rIL-15 +/− PGE2 resulted in a dose dependent decrease in cytotoxicity (Cr-51 labelled K562s) and decreases in IFNγ production. The authors concluded that PGE2 treatment was interfering with the process of common γ-chain (γ_c_-chain) translocation to the surface of NK cells, a protein used by many cytokine binding and signalling (IL-2, IL-4, IL-7, and IL-9) pathways. It is possible that the rise in IL-6 and PGE2 post-surgery is interfering with NK cell cytokine signalling mechanisms, however there has been no studies to prove this yet.

Post-operative TGF-β levels has been associated with increased metastasis to lymph nodes after surgical prostate removal in patients [55]. TGF-β is known to suppress NK cell activity, function and responsiveness to various activating cytokines [56,57,58]. Of the different signaling cascades affected by TGF-β, inhibition of the mammalian target of rapamycin (mTOR) pathway has been directly implicated in suppressing anti-tumour efficacy of NK cells [59]. In particular, increased engagement of TGF-β with its receptor on NK cells reduces proliferation, metabolism and cytotoxicity of NK cells by countering IL-15 or IL-2 mediated mTOR activation. In cancer patients, TGF-β has been shown to drive downregulation of the activating NKG2D receptor, resulting in unregulated tumour proliferation [60,61]. In hepatocellular carcinoma patients, POD30 NKG2D expression on CD56^dim^ NK cells was significantly lower in patients which had recurred compared to those that remained recurrence free during a two-year follow-up [10]. Notably, there was a significant negative correlation of NKG2D^+^CD56^dim^ NK cells to serum concentrations of TGF-β (*r* = −0.640, *p* = 0.001) and soluble MICA (*r* = −0.487, *p* = 0.016). Since IL-6 drives TGF-β production from various cell types and TGF-β itself may enhance IL-6 release, a resulting feedback loop may perpetuate post-surgical immune suppression [62,63,64]. Furthermore, platelets [65] and shear stress [66] have been reported to increase TGF-β. In the post-surgical environment an increase in TGF-β can cause NK cell dysfunction. Interestingly, Zhao et al. [67] has shown that genetically modifying NK cells to express a dominant-negative TGF-β type II receptor can render NK cells insensitive to the suppressive effects of TGF-β. Utilizing this genetic modification could potentially increase the effectiveness of investigational peri-operative adoptive NK cell therapies.

### 4.2. Analgesics, Anaesthetics, Blood Transfusions and Their Effect on Post-Operative NK Cell Function

Analgesics, anaesthetics and blood transfusions have also been associated with immune suppression in the post-operative period. In neck surgery patients sedated for 48 h post-surgery, it was observed that in those receiving fentanyl (a synthetic opioid analgesic), NK cells collected on POD 1 demonstrated reduced cytotoxicity ex vivo compared to those receiving the non-steroidal anti-inflammatory drug (NSAID), flurbiprofen [68]. Preclinical studies in murine and rodent models have demonstrated that infusion of fentanyl significantly reduces lymphocyte proliferation and NK cell cytotoxicity [69,70,71]. Opioid-mediated suppression of immune cells may be attributed to direct engagement of opioid receptors on lymphocytes modulating downstream MAPK and cAMP signaling or via indirect effects on TLR-4 signaling [72,73,74,75]. However, in healthy volunteers, while morphine was shown to reduce NK cell cytotoxicity, fentanyl boosted NK cell activity suggesting that effects of opioids may differ in surgical and non-surgical patients [76,77].

Peri-operative blood transfusions are routinely used during cancer surgeries, however, many retrospective studies have reported increased recurrence, post-operative infection rates, and morbidity as a result of blood transfusions [78,79,80]. Gharehbaghian et al. demonstrated that use of autologous salvaged blood, instead of allogenic blood for transfusion, increased the proportion of NK cell precursors and IFNγ production post-surgery [81]. However, there was no difference in autologous vs. allogenic blood transfusion in NK cell cytotoxicity in Takemura’s study [82]. These results may be explained by the fact that blood transfusions themselves will result in immunosuppression. Therefore, blood transfusions should be minimized to reduce the amount of post-surgical NK cell suppression.

### 4.3. The Post-Operative Hypercoaguable State Shields Cancer Cells and Blocks NK Cell Cytotoxicity

Surgical trauma activates platelets and leads to a hypercoagulable state which lasts for up to a month following surgery [83], which increases the risk of venous thromboembolic events (VTE). In cancer patients, VTE are associated with a worse cancer-specific survival and a hypercoagulable state is associated with the formation of metastases [84]. The mechanism appears to be platelet mediated P-selectin binding to tumour cells, which impairs NK cell mediated tumour cell destruction. Anticoagulants, such as low molecular weight heparin (LMWH), are considered the standard of care for preventing post-operative VTE but the duration of treatment is controversial [85]. Interestingly, LMWH has also been associated with an improved cancer survival and has been shown in numerous preclinical studies to have anti-metastatic properties, because it can block P-selectin mediated binding. Research from our laboratory has shown that post-operative hypercoagulation may responsible for the formation of post-operative metastases by inhibiting NK cell clearance of tumour cells secondary to peri-tumoural platelet and fibrin clots [8]. We report that using peri-operative LMWH in murine models of surgical stress effectively restored NK cell cytotoxicity and prevented post-operative metastatic disease. A Phase 3 trial using peri-operatively LMWH in CRC surgery patients is currently underway (NCT01455831).

### 4.4. Decreased Mitochondrial Membrane Potential (ΔΨm) Increases NK Cell Susceptibility to Apoptosis

Increased susceptibility to apoptosis in adaptive immune lymphocytes have been reported to occur in the days after surgery [43]. However, a mixture of studies report conflicting data on the number of circulating NK cells following surgery. While some reports (including our own) observe no substantial changes in circulating NK cell populations following a major procedure, others have reported a decrease on POD1 [17,39,86]. This discrepancy may be due to study design differences in PBMC assessment times, NK cell identification, and the type of surgical operation. The phenomenon of decreased circulating peripheral blood lymphocytes following surgery may be indicative of extravasation in response to the surgical site pro-inflammatory signals or of increased apoptosis of lymphocytes. Takabayashi et al. [87] conducted a study of five different surgical procedures with varying levels of intensity and complexity to look at the changes in mitochondrial membrane potential (ΔΨm), an early marker of apoptosis. This was performed in a number of PBL cell types (CD3^+^ T cells, CD56^+^ NK cells, and CD19^+^ B cells). They showed that major cancer surgeries (intrathoracic esophagectomies and hepatic resection of liver metastases) resulted in the most dramatic decreases in ΔΨm, and that NK cells suffered the greatest drop in membrane potential. When correlating ΔΨm to NK cell cytotoxicity after surgery, they reported that greater changes in ΔΨm correlated with greater decreases in NK cell cytotoxicity (*r* = 0.825; *p* = 0.0003) [87]. Membrane potential was significantly correlated with plasma noradrenaline levels (*r* = −0.578; *p* = 0.0008). Lastly, this article highlights that minimally invasive surgeries (laparoscopic cholecystectomy) had the least effect on NK cell membrane potential which may explain why some reports did not observe a decrease in NK cell numbers. Therefore, the surgical stress environment may be promoting pro-apoptotic mechanisms in PBL which render them ineffective following surgery.

### 4.5. Correlating Post-Operative NK Cell Suppression to the Degree of Surgical Stress

In a study profiling immune subsets on patients undergoing different minor and major surgeries, significant decreases in lymphocyte populations was observed with major surgeries resulting in the most decrease and slowest recovery to basal levels [88]. NK cells were found to be lower post-operatively along with T cells and NKT cells and this effect was more pronounced in patients undergoing major surgery versus minor surgeries. Similarly, in non-small cell lung cancer patients NK cells were found to be significantly lower in patients undergoing open thoracotomy (major) versus video-assisted thoracoscopic lobectomy (minor) [89].

Animal models assessing the effect of minimally invasive surgery vs. open surgery have shown considerable differences between the two procedures when reporting NK cell cytotoxicity [12,90]. However, the same has not held true when assessing data collected from clinical trials [91]. Efforts to reduce surgical stress (e.g., opting for less invasive surgical procedures such as laparoscopies or optimizing anaesthetic and analgesic regimens) have not consistently shown reductions in post-operative recurrence or metastases [92,93]. However, the differences between laparoscopy vs. open laparotomies become more evident when looking at cancer surgeries occurring at later disease stages. Assessing disease free survival (DFS) in Stage III CRC patients, Bonjer et al. [93] reported that DFS was 64.9% after laparotomy (*n* = 233) compared to 52% or open surgeries (*n* = 127).

## 5. Post-Operative Myeloid Derived Suppressor Cells and Natural Killer Cell Dysfunction

Myeloid derived suppressor cells (MDSCs) are a heterogeneous population of immature myeloid cells that are identified by their ability to suppress the immune system. While these suppressive cells were initially described in relation to the tumour microenvironment in cancer, their existence is seen throughout acute inflammatory states such as bacterial infection [94], trauma [95] and surgery patients [96]. Specific MDSC biomarkers are still being defined in humans MDSC populations as there are very large inter-laboratory differences in reporting MDSCs [97]. The most recent guidelines for identifying MDSCs are outlined in Bronte et al. [98] which lists many descriptions of phenotypic, functional, and biochemical/molecular attributes that must be met to justify a MDSC classification. In humans, phenotypic characterizations of MDSCs fall under three myeloid specific (CD11b^+^ or CD33^+^), lineage negative (CD3^−^CD56^−^CD19^−^) subsets: (1) CD14^+^CD15^−^ monocytic MDSCs (M-MDSCs); (2) CD14^−^CD15^+^ polymorphonuclear MDSCs (PMN-MDSCs) or; (3) CD14^−^CD15^−^HLA-DR^−^ early/immature-MDSCs (eMDSCs) [98]. After determining which MDSC subtype accumulates in a certain pathological setting, a functional test assessing their suppressive capacity must be shown to classify them as a bona fide MDSC. Most functional reports on MDSCs use an in vitro co-culture of MDSCs and T cells stimmed with anti-CD3/anti-CD28 and assess T cell proliferation, however, MDSCs are also able to suppress NK cell cytotoxicity and IFNγ production [99,100,101].

Since MDSCs are associated with a tumour microenvironment, cancer surgery patients have a pre-existing pool of suppressive MDSCs which Yuan et al. [102] reported to be a potential prognostic marker for DFS and local recurrence free survival (LRFS) after curative surgery in 64 rectal cancer patients. MDSCs in this study were defined as HLA-DR^−^Lin^−^CD33^+^CD11b^+^ cells with the ability to suppress T cell proliferation [102]. The MDSC population in these surgery patients increased from 3.89% before surgery to 7.1% on POD7 before contracting to 4.39% on POD14 and finally to 2.21% on POD21—which is below pre-operative levels. Importantly, they showed that in patients with higher numbers of MDSCs before surgery as well as on POD21 after surgery DFS and LRFS were significantly decreased (*p* < 0.05). In our studies we have seen a more dramatic increase in surgery induced MDSCs going from 12.4% before surgery to 37.3% on POD1 (*n* = 12, various cancer surgeries, CD33^+^CD14^+^HLA-DR^low/neg^).

Many factors contribute to the generation of MDSCs. Prostaglandins, COX2, IL-6, IL-4, VEGF, GM-CSF and the process of emergency myelopoiesis could contribute to the expansion of not-fully mature myeloid cells into the blood stream to restore homeostasis in lieu of surgical stress [103]. The increase in post-operative MDSCs reported by Wang et al. [103] had increased VEGF expression. Transferring MDSCs from preoperative and post-operative patient blood samples into nude mice with the human lung cancer cell line, A549, demonstrated that post-operative MDSCs contributed to angiogenesis and tumour growth in these mice. This group later reported that M-MDSCs (CD14^+^) increased significantly following surgery and high M-MDSC numbers (>0.435 × 10^9^/L, *n* = 31) on POD7 resulted in a significant reduction in recurrence free survival (*p* = 0.039) [104]. Therefore, MDSCs in the post-operative period may effect tumour growth by altering non-immunologic mechanisms.

MDSCs can suppress immune cells through multiple context dependent mechanisms. M-MDSCs have upregulated expression of inducer of nitric oxide synthase enzyme which can produce large amounts of NO that can lead to nitration of T cell receptor and suppression. PMN-MDSCs have been more reported to use arginase-1 dependent mechanisms of suppression whereby the depletion of arginine, a conditionally essential amino acid, can reduce CD3ζ chain expression and proliferation of T cells. Both of these suppressive pathways rely on the bioavailability of the common amino acid substrate, arginine. In addition to the arginine metabolising mechanisms MDSCs can produce large amounts of reactive oxygen species (ROS), reduce cysteine, and is involved in the recruitment of Tregs [104]. Given their broad suppressive mechanisms and abundance in the post-operative period, surgery induced MDSCs may have a large stake in NK cell dysfunction.

### Post-Operative Myeloid Derived Suppressor Cells Can Suppress NK Cell Function

An early study by Uchida and colleagues showed that PBMCs isolated from 16/21 breast cancer patients undergoing radical mastectomies had a reduction in NK cell-specific killing that was significantly less on POD7 compared to pre-operation levels. The number of large granular lymphocytes did not change after surgery but culturing post-operation PBMCs for 24 h in monocyte depleted conditions recovered NK cell-specific cytotoxicity while undepleted cultures still suffered a > 50% drop in cytotoxicity [105]. Using normal NK cells cultured with monocytes from surgery patients, but not conditioned media of monocytes, suppressed NK cell killing and they concluded that the emergence of a suppressive myeloid cell in response to surgical stress caused NK cell dysfunction [105]. Decades later, it is possible that the suppressive cells described by Uchida et al. were in fact surgery-induced MDSCs.

We have shown that surgery induces MDSCs to accumulate (up to four fold) and suppress NK and T cells in murine models and human patient blood samples [7,106]. There is a paucity of information regarding the mechanisms of NK cell suppression by MDSCs. It is evident from the studies on MDSC mediated NK cell suppression that the suppressive mechanisms vary depending on the pathological context. During pox or adenoviral infections, MDSCs produce large amounts of ROS which result in NK cell suppression [107,108]. Interestingly, in chronic Hepatitis C viral infection, NK cell activation was suppressed in a contact- and ROS-independent mechanism [101]. In hepatocellular carcinoma patients, MDSCs suppress NK cells through contact dependent inhibition mediated by NKp30 [100]. To complicate things further, in a murine model of orthotopic liver cancer, membrane bound TGF-β1 on MDSCs in tumour bearing mice suppressed NK cells via a contact-dependent mechanism [109]. These conflicting reports show the heterogeneous mechanisms and context dependent nature of MDSC mediated NK cell dysfunction. The mechanism of MDSC mediated NK cell dysfunction following surgery remains unknown, however they may be closely related to the arginine metabolising properties of MDSCs.

## 6. Nutritional Deficiencies Following Surgery and Natural Killer Cell Dysfunction

**Figure 1:**
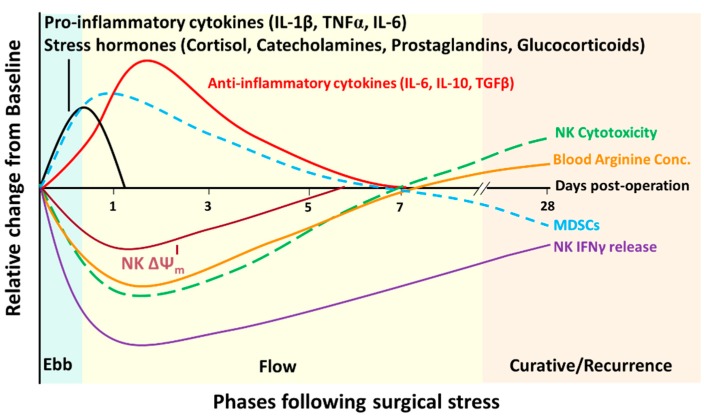
Aligning NK cell dysfunction with the ebb and flow of cancer surgery. Immediately following surgical incision, a robust pro-inflammatory cytokine and stress response ensue marking the “ebb” phase of cancer surgery. The ebb phase subsides within 12 h due to the release of anti-inflammatory cytokines, hypermetabolism, increased cardiac output, and surgery-induced MDSCs which collectively composes the “flow” phase. During the flow phase NK cell cytotoxicity, cytokine production and mitochondrial membrane potential (ΔΨm) are at its lowest (POD1) but cytotoxicity and ΔΨm gradually normalize to pre-operative levels by POD7. IFNγ production, however, remains affected for up to 28 days after surgery. The curative phase is based on the hypothesis that following curative cancer surgery, the patient will have the benefit of being free of primary tumours which will lead to an increase in NK cell function and less MDSCs. Recurrence will occur if minimal residual disease or dormant metastatic growths establish themselves during the flow phase of reduced anti-tumour surveillance after surgery.

The natural response to traumatic injury was described by Cuthbertson over 70 years ago to be composed of two phases (similar to the pro- and anti-inflammatory response to surgery). The ebb and flow model (Figure 1) was presented which describes the “ebb” phase as an acute, hypometabolic period lasting for only up to 12 h which ensues to conserve energy [110]. The “flow” phase following traumatic injury or surgical stress is associated with hypermetabolism which generally persists for seven days but can last for up to three weeks after severe injury [110,111,112]. Increased oxygen consumption, body temperature, tachycardia, insulin resistance, hyperglycemia, protein catabolism, and lipolysis result in a high nutritional demand for post-operative cellular processes during the flow phase [110,112,113]. Thus, basal metabolic rates are increased following surgery to restore homeostasis in the patient. These physiological responses are important initially but prolonged nutrient deficiency can increase post-operative complications and lengthen hospital stay, and should be counteracted peri-operatively [112]. Specific nutrients have been incorporated into peri-operative nutritional regimens with a specific focus on enhancing post-operative immune function and have therefore been called “immunonutrients”. Glutamine, arginine, and omega-chain fatty acids, are effective in this. The beneficial effects of supplemental arginine around the time of surgery was reported to have a substantial impact on reduced infectious complications and hospital length of stay in a meta-analysis of *n* = 28 and *n* = 29 randomized clinical trials, respectively [114].

### 6.1. Arginine Deficiencies Following Surgery and NK Cell Dysfunction

Arginine is a conditionally essential amino acid which has been known to suffer a 50% drop in plasma concentrations immediately after trauma or surgical stress [115,116]. Coupling this with the fact that cancer patients have lower plasma arginine concentrations than healthy individuals means that the arginine deficiency in the aftermath of cancer surgery drops well below normal physiological concentrations (100–150 μM plasma arginine) [117,118,119]. In mice, increasing systemic arginine has been shown to slow tumour progression and prolong survival in a 4T1 breast cancer model [120] and in a SW480 human CRC model [121]. Ex vivo human studies have shown that arginine treatment of NK cells and lymphokine activated killer cells from healthy donors also increased their cytotoxicity against Cr-51 labelled Daudi cells [122]. Furthermore, this study showed a significant increase in CD56^+^ cells following oral arginine administration at 30 g/day for three days in healthy subjects.

The functional role of arginine for NK cell cytotoxicity has been demonstrated decades earlier by Xiao et al. [123]. They showed that primary NK cells had a 70% reduction in their cytotoxicity potential when cultured in “deficient media” (devoid of amino acids, serum, pyruvate, vitamins) compared to “complete media” (RPMI 1640, 10% FCS, penicillin, and streptomycin). Importantly, adding L-arginine (1mM) back into the NK cell cultures restored NK cell cytotoxicity while adding various other components back into the deficient media yielded minimal recovery in NK cell cytotoxicity. Even adding everything back into the deficient media except for arginine did not increase NK cell killing above the levels of only adding arginine to the deficient media. This experiment provided initial evidence that NK cells rely on adequate arginine levels for proper function. Oberlies et al. [124] confirms this postulation by showing that NK cell function (proliferation and IFNγ production) was not lost when incubated in tryptophan depleted environments but severely affected after culturing in arginine-depleted media.

In the absence of amino acids the mTOR signaling pathway is inhibited which leads to a decrease in protein translation and cell proliferation. Goh et al. [101] showed that human NK cells cultured in arginine-deficient media for two days are not affected in their viability nor granzyme B production, but were significantly reduced in IFNγ production. They also showed that there was a decrease in mTOR phosphorylation and its downstream target, 4EBP1, compared to NK cells grown in complete media. These experiments suggest that arginine depletion is causing a reduction of post-transcriptional processes within NK cells which may be related to the mTOR pathway. Oberlies et al. [124] showed earlier that IFNγ transcript levels were not different after 24 h IL-12/IL-18 stimulation in either arginine deplete or arginine sufficient media by real-time RT-PCR quantification. They assessed another amino acid sensing pathway, the activation of general control non-derepressible 2 (GCN2) kinases. The depletion of arginine can lead to an increase in uncharged tRNA molecules which activates GCN2 kinase to phosphorylate the eukaryotic initiation factor-α (eIFα) which inhibits eIFβ, thus stopping protein translation. This has been shown to occur in T cells deprived of arginine, while GCN2 knockouts are not affected by arginine deprivation because they have lost their ability to sense amino acid deprivation [125,126]. Interestingly, Oberlies et al. showed that NK cell translation of IFNγ is not regulated by GCN2 kinase as there was no increase in phosphorylated GCN2 when cultured in arginine free conditions. They also showed that tryptophan depletion did not cause NK cell dysfunction which implies that the mechanism is specific to arginine and not a general amino acid deprivation response [124].

## 7. Strategies to Improve Post-Operative NK Cell Function through Investigational Peri-Operative Therapies

While there are a number of clinical trials assessing peri-operative therapies, only a handful have experimental endpoints measuring NK cell function. These can be categorized into trials designed to (1) enhance immune function pre-operatively, (2) prevent immune suppression post-operatively, or (3) investigate non-immune augmenting peri-operative therapies (Table 1).

Enhancing NK cell function pre-operatively may be an effective strategy to improve patient outcome following surgery. Peri-operative adoptive transfer of NK cells is being researched which could circumvent the immunosuppression of NK cells by surgery induced factors. It will be interesting to see whether NK cells transferred after surgery will become suppressed due to suppressive factors released after surgery or if NK cells needed to be present at the time of surgical trauma. Our research group is using an immunonutritional supplement (INergy) enhanced in arginine content in a Phase Ib clinical trial in CRC surgery patients with the primary endpoint being correlative assays of NK cell function (NCT02987296). As discussed previously (Section 6.1), we hypothesize that boosting pre-operative arginine levels will reduce post-operative NK cell dysfunction associated with arginine deprivation. Additionally, we have also determined that peri-operative influenza vaccination is able to attenuate metastases and increase post-surgical NK cell function in murine models [132]. Additionally, phosphodiesterase-5 (PDE5) inhibitors can effectively regulate arginase-1 activity and MDSC accumulation [133]. Combining pre-operative vaccination with peri-operative PDE5 inhibitors has shown promising preclinical results [134] which has prompted us to initiate a Phase 1 study of peri-operative administration of Tadalafil (a PDE5 inhibitor) in combination with influenza in CRC surgery patients (NCT02998736).

Administration of low-dose IFNα or IL-2 pre-operatively has shown clinical efficacy in numerous clinical trials assessing NK and T cell suppression [127,128,129,130,135,136,137]. In all of the studies assessing non-specific immune stimulating IL-2 therapies, adverse events were limited to pyrexia (grade I-III) and was overall well tolerated. A cohort of 86 stage II and III CRC patients were randomized to receive short-term IL-2 cytokine therapy or a placebo before their open surgery. This study showed that treating with IL-2 two times a day for three consecutive days prior to surgery resulted in significantly less recurrences compared to the placebo group (21.4% vs 43.1%, *p* = 0.03) at the median follow up time of 54 months [137]. A separate randomized clinical trial showed that pre-operative IL-2 treatment also significantly improved post-operative lymphocyte and NK cell numbers compared to placebo in Stage IV CRC patients [130]. Those that received pre-operative IL-2 also had a survival benefit when assessed at one year post-operation. Lastly, a Phase II trial of 120 renal cell carcinoma surgery patients had a significant improvement in five-year progression free survival (74% vs. 62%, *p* = 0.02) when given IL-2 pre-operatively [129]. Therefore, evidence from these studies show that it is possible to mitigate surgery induced immunosuppression and improve patient outcomes by giving pre-operative immunotherapies to enhance NK and immune cell activity.

The majority of investigational peri-operative clinical trials have a main focus on preventing immune suppression by understanding the use of various anaesthetics and analgesics (described in Section 4.2). However, treatment during the peri-operative period is accompanied by a few hurdles which must be considered when translating preclinical findings to clinical trials. While each cancer patient’s medication requirements differ considerably from another, certain drug classes are suspended during the peri-operative period. NSAIDs such as aspirin, for example, are generally stopped seven days before surgery as they can bind irreversibly to COX enzymes which would prevent the synthesis of clotting factors, important for regulating vasodilation and coagulation. However, the specific COX2 inhibitor, etodolac, can be used peri-operatively because it will not lead to vasodilation. Recent results from a randomized Phase II clinical trial assessing multiple tumour and circulating biomarker parameters report that etodolac in combination with the β-blocker, propranolol, is well tolerated and effective in mitigating the prometastatic effects of surgical stress in early stage breast cancer patients (NCT00502684) [131]. The authors report that this combination of peri-operative drugs decreased molecular biomarkers and transcriptional pathways (GATA-1, GATA-2 and EGR3) of epithelial-to-mesenchymal transition, reduced pre- and post-operative serum IL-6 and CRP levels, reduced the number of tumour infiltrating monocytes and also increased NK cell activation markers (CD11a) following surgery. The promising results from this study warrants the investigation of the long term outcomes of cancer surgery patients given peri-operative COX2 inhibitor and β-blocker therapy.

Finally, peri-operative strategies that do not directly target immune function (non-immune augmenting) are being investigated (such as the use of LMWH). Furthermore, surgical practice is now evolving to opt for the least invasive procedures when treating cancer patients and hospitals are adopting multifaceted guidelines for peri-operative care, such as “Enhanced Recovery after Surgery” (ERAS). ERAS components differ slightly depending on the specific type of surgery, but all ERAS programs aim to improve the quality of patient recovery. Fundamental components of ERAS include nutritional support and optimal opioid-sparing anaesthetics and local analgesics [138,139,140]. Therefore, the outcomes of these clinical trials will yield valuable information on immune and NK cell function which could inform future iterations of ERAS.

## 8. Conclusions

The physiological changes that occur after surgery is hypothesized to be the result of conserved evolutionary traits which have been passed down to prolong survival after unplanned traumatic injuries. However, these changes may not be necessary when surgical stress occurs electively in a sterile environment. The immediate post-operative period represents a critical window for therapeutic intervention which has been largely overlooked. Research into this phenomenon has begun to clarify the order of events which occur and their potential impact on NK cell function (Figure 1). This presents us with an opportunity to target these post-operative changes to improve the lasting therapeutic benefit of curative cancer surgery. Table 1 lists the exciting clinical trials that are underway with a focus on improving NK cell function before surgery, or preventing their dysfunction after surgery. We hypothesize that to fully realize improved NK cell function following surgical stress a concerted effort of peri-operative immune therapies and ERAS protocols will need to be combined. This will ultimately improve overall patient recovery and recurrence free survival and lead to truly curative cancer surgeries.

## Figures and Tables

**Table 1:** Clinical trials specifically assessing NK cell function after peri-operative intervention during cancer surgery as a primary or secondary experimental endpoint.

Peri-Operative Target	Trial ID, Phase	Intervention	Study Title
**Enhance Immune Function Trials**			
Adoptive Cell Transfer	NCT02725996, Phase II	NK cells	By Using Adoptive Transfer of Autologous NK Cells to Prevent Recurrence of Hepatocellular Carcinoma After Curative Therapy
Cytokine Therapy	[127]	IFNα	Peri-operative IFN-alpha to avoid surgically induced immune suppression in colorectal cancer patients
	[128,129] Phase II	IL-2	Peri-operative immunomodulation with interleukin-2 in patients with renal cell carcinoma
	[130]	IL-2	Preoperative interleukin-2 subcutaneous immunotherapy may prolong the survival time in advanced colorectal cancer patients.
Innate Immune Stimulation and PDE5 Inhibition ^a^	NCT02998736, Phase I	Cialis	Trial of Peri-operative Tadalafil and Influenza Vaccination in Cancer Patients Undergoing Major Surgical Resection of a Primary Abdominal Malignancy (PERIOP-04)
Immuno-nutrition	NCT02987296, Phase Ib	Dietary Supplemental Arginine	Peri-operative Immunonutrition in Colorectal Cancer Patients Undergoing Abdominal Surgery (PERIOP-02)
**Prevent Immune Suppression Trials**			
Anaesthetic/ Analgesic	NCT01841294, Phase IV	Lidocaine	NK Activity Modulation Induced by Intravenous Lidocaine During Colorectal Laparoscopic Surgery
	NCT01367418, Phase III ^b^	Epidural (Bupivacaine; Ropivacaine; Sufentanil)	Effects of Anesthetic Technique on Immune and Inflammatory Systems Following Radical Prostatectomy (AIMS)
	NCT01929915	Epidural	Analgesia and Pancreatic Cancer Surgery
	NCT02326727	Epidural Ropivacaine	Influence of Epidural Analgesia on Natural Killer Cell (NK) Activity After Colonic Cancer Surgery
	NCT02567942	Propofol vs. Sevoflurane	Assessment of the Anesthetic Effect on the Activity of Immune Cell in Patient With Colon Cancer
	NCT02896413	Dexmedeto-midine	The Effects of Peri-operative Dexmedetomidine Administration on Immune Suppression and Outcomes in Patients With Uterine Cancer Undergoing Radical Resection
	NCT03109990	Dexmedeto-midine	Impact of Dexmedetomidine on Breast Cancer Recurrence After Surgery
Complementary Therapy	NCT02620033 ^c^	Yoga Therapy	Impact of Yoga As Complementary Therapy in Patients Undergoing Radical Prostatectomy
COX2 Inhibitor	NCT00502684 Phase II [131]	Propranolol, etodolac	Peri-operative Administration of COX 2 Inhibitors and Beta Blockers to Women Undergoing Breast Cancer Surgery
**Non-Immune Augmenting Trials**			
Hyper-coagulation	NCT01455831, Phase III	Tinzaparin	Extended Peri-operative Tinzaparin to Improve Disease-free Survival in Patients With Resectable Colorectal Cancer (PERIOP-01)
Exploratory	NCT02661776	None	The Change of NK Cell Activity After Head and Neck Cancer Surgery

^a^ Trial can also be categorized in 2); ^b^ Trial is completed; ^c^ Trial has been discontinued.

## Abbreviations

Cr-51	Chromium-51
CRC	Colorectal cancer
CRP	C-reactive protein
DAMPs	Danger associated molecular patterns
DFS	Disease free survival
ERAS	Enhanced recovery after surgery
IL	Interleukin
KIRs	Killer Ig-like receptors
LMWH	Low molecular weight heparin
LRFS	Local recurrence free survival
LRP	Laparoscopic radical prostectomy
MDSC	Myeloid derived suppressor cell
MIC	MHC-Class I polypeptide-related sequence
NCR	Natural cytotoxicity receptors
NK	Natural Killer
NSAID	Non-steroidal anti-inflammatory drug
ORP	Open radical prostectomy
OS	Overall survival
PBL	Peripheral blood lymphocytes
PBMC	Peripheral blood mononuclear cells
PDE5	Phosphodiesterase-5
PFS	Progression free survival
PGE2	Prostaglandin E2
POD	Post-operation day
PRRs	Pattern recognition receptors
ULBP	UL16 Binding proteins
VTE	Venous thromboembolic events
ΔΨm	Mitochondrial membrane potential
